# A survey of ATRIPLA use in clinical practice as first-line therapy in HIV-positive persons in Europe

**DOI:** 10.1007/s15010-014-0630-4

**Published:** 2014-06-06

**Authors:** A. Mocroft, P. Reiss, A. Rakhmanova, D. Banhegyi, A. N. Phillips, S. De Wit, M. Ristola, J. D. Lundgren, J. Grarup, O. Kirk, for EuroSIDA in EuroCOORD

**Affiliations:** 1 Department of Infection and Population Health, University College London, Rowland Hill St, London, NW3 2PF UK; 2 Academisch Medisch Centrum bij de Universiteit van Amsterdam, Amsterdam, The Netherlands; 3 Stichting HIV Monitoring, Amsterdam, The Netherlands; 4 Medical Academy Botkin Hospital, St Petersburg, Russia; 5 Szent Lásló Hospital, Budapest, Hungary; 6 Saint-Pierre Hospital, Brussels, Belgium; 7 Helsinki University Central Hospital, Helsinki, Finland; 8 Copenhagen HIV Programme, University of Copenhagen, Copenhagen, Denmark; 9 Department of Infectious Diseases, Rigshospitalet, Copenhagen, Denmark

## Abstract

ATRIPLA is licensed for use only in HIV-positive persons whose viral loads <50 for ≥3 months. We investigated the use of ATRIPLA as first-line antiretroviral therapy (ART) in EuroSIDA using a web-based survey performed in Autumn 2012. 96/112 clinics (85.7 %) completed the survey. Recommendations when initiating first-line ART was TRUVADA plus efavirenz in 36 (37.5 %), ATRIPLA in 35 (36.5 %), a different first-line regimen in 12 clinics (12.5 %), and no recommendation in 7 clinics (7.3 %). ATRIPLA was commonest in Northern (15/21 clinics; 71.4 %), and least common in Eastern Europe (2/31 clinics; 6.5 %; *p* < 0.0001). Over one-third of the participating clinics in this survey were using ATRIPLA as first-line antiretroviral therapy, despite EMA recommendations.

## Background

ATRIPLA is a once-daily fixed-dose combination of a non-nucleoside reverse transcriptase inhibitor (NNRTI) efavirenz (EFV; 600 mg), and the nucleoside reverse transcriptase inhibitors (NRTI) tenofovir disoproxil fumarate (TDF; 300 mg) and emtricitabine (FTC; 200 mg), which have potent activity against HIV-1 infection [1, 2]. The European Medicines Agency (EMA) has licensed ATRIPLA for use only in HIV-positive persons whose levels of HIV in the blood (viral loads) have been below 50 copies/ml for more than 3 months on their current HIV treatment combination [3]. The reasons for the current EMA labelling is that intake of tenofovir is normally with food, while ATRIPLA is usually taken at night prior to bed and therefore in a semi-fasting state [3, 4]. Demonstration of non-inferior viral outcome from use of ATRIPLA versus TRUVADA + efavirenz would require a sizable phase IV trial, which has not yet been performed. Despite this, a sizeable proportion of persons initiating antiretroviral therapy are believed to start ATRIPLA and not on the individual components as recommended by the label. It is important to understand the extent to which centres treating HIV-positive individuals adhere to EMA recommendations in general. The aim of this survey was to assess ATRIPLA use as first-line antiretroviral therapy in daily clinical management of HIV-infected persons from across Europe.

## Methods

The EuroSIDA study was initiated in 1994, and is a prospective observational cohort study of more than 18,000 HIV-positive persons followed in 112 hospitals in 33 European countries, plus Israel and Argentina (details at http://www.chip.dk). A cross-sectional web-based survey of HIV clinics participating in EuroSIDA was used to investigate the use of ATRIPLA, or its components, as first-line antiretroviral therapy in HIV-infected persons in diverse clinical settings in Europe. The survey was completed as an electronic survey using REDCap™, in agreement with the REDCap Consortium, Vanderbilt University included information collected from treating physicians about their normal department policy for treatment of HIV-infected persons initiating a first-line antiretroviral therapy regimen containing ATRIPLA as a fixed-dose once-daily combination tablet, or its individual components in Europe. For the purposes of descriptive analysis, EuroSIDA has been divided into four geographical regions, as previously described—South, Central West, North, East and Argentina [5]. For the present analysis, Argentina (only one clinic participating in EuroSIDA) has been merged with data from Southern Europe.

## Results

A total of 96/112 clinics (85.7 %) completed the survey. Summary characteristics of those who completed or did not complete the survey are shown in Table 1, with few differences between participating and non-participating sites. Clinics with persons with a higher median CD4 count at recruitment were less likely to participate [adjusted odds ratio (aOR) 0.51 per 50/mm^3^ higher median CD4; 95 % confidence interval (CI) 0.32–0.81, *p* = 0.0043)], while clinics with a higher median proportion on cART were more likely to participate (aOR 1.34 per 10 % higher; 95 % CI 0.99–1.80, *p* = 0.057), as were clinics with a later median date of enrolment in EuroSIDA (aOR 1.28 per year later; 95 % CI 1.02–1.60, *p* = 0.030). Of note, there were no differences between regions in terms of participation in the survey.

**Table 1:** Comparison of summary statistics between participating and non-participating centres

	Excluded		Included		*p*
	*N*	%	*N*	%	
*N*	16	14.3	96	85.7	
Region					
South	7	24.1	22	75.9	0.35
Central West	3	12.0	22	88.0	
North	3	12.5	21	87.5	
East	3	8.8	31	91.2	

	Median	IQR	Median	IQR	
Male gender	74.9	65.1–85.5	75.0	67.2–81.0	0.61
Age (years)	35.6	33.8–38.1	36.6	33.9–39.3	0.40
White race	94.0	88.7–98.2	94.6	83.7–99.0	0.91
Homosexual	34.4	16.9–63.8	36.2	19.4–54.7	0.65
IDU	11.8	4.2–38.0	20.0	6.6–38.5	0.41
Heterosexual	26.3	17.3–42.0	27.5	20.0–36.3	0.76
Prior AIDS	23.4	14.8–28.1	27.5	19.2–33.5	0.31
Started cART	32.3	10.5–63.7	50.4	32.2–72.1	0.078
ARV naïve	31.0	20.9–49.9	25.3	17.1–35.3	0.17
VL <400	57.0	44.0–67.1	59.6	46.0–70.8	0.44
CD4 (/mm^3^)	372	224–465	339	257–400	0.55
VL (log_10_cp/ml)	2.4	1.7–2.9	2.6	1.7–3.0	0.57
Enrollment (month/year)	3/99	7/94–3/04	1/02	2/97–5/06	0.16

Figures in tables are based on summary statistics from the main EuroSIDA clinical database, and are not part of the data collected in the survey. The proportion of, for example, males, within each centre has been extracted from the main database, and the figure in the table is the median of these proportions. Similarly, the median CD4 at enrolment within each centre has been extracted, and the figure in the table represents the median of these medians

The median number of persons cared for in the clinics surveyed was 1,200 [interquartile range (IQR 665–2,150)], with no significant variation across the regions surveyed. The median proportion on cART was 80 % (IQR 70–90 %), with the highest proportion in Central West and Northern Europe (both median 85 %, IQR 80–90 %), followed by Southern Europe (median 80 %, IQR 70–90 %) and the lowest proportion in Eastern Europe (median 60 %, IQR 35–75 %, *p* < 0.0001).

**Fig. 1:**
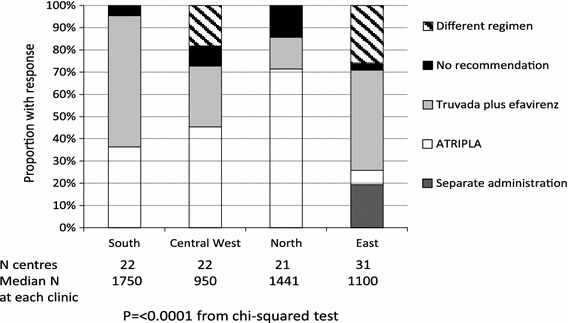
Current recommendations for first-line cART

36 clinics (37.5 %) indicated that the current recommendation when initiating first-line antiretroviral therapy was that tenofovir and emtricitabine are administered as one tablet, with efavirenz administered separately; 35 clinics (36.5 %) indicated that ATRIPLA was the current recommendation; 12 clinics (12.5 %) indicated that they usually use a different first-line regimen and 7 clinics (7.3 %) indicated that the decision was up to the treating physician with no general recommendation. Six clinics (6.3 %), all from Eastern Europe, said the three components were administered separately. There were significant differences between regions (Fig. 1) (*p* < 0.0001). Among the six clinics responding that the three components were administered separately, one was due to a local decision and financial considerations, one was due to national guidelines, one due to a clinical decision, and one for purely financial reasons. Two clinics stated it was because ATRIPLA was not routinely available in their country (Hungary and Romania).

Of the 35 clinics which used ATRIPLA as the first-line regimen, 18 (51.4 %) stated it was a local decision, 15 (42.9 %) due to national guidelines, 8 (22.9 %) due to European guidelines and 4 (11.4 %) for other reasons. There was some overlap between reasons, as clinics were allowed to indicate more than one choice; two clinics indicated it was due to both national guidelines and a local decision, six clinics stated it was due to national and European guidelines and two due to local decisions and European guidelines. There were no significant regional differences in the proportion of clinics indicating ‘local decision’ as reason for choosing ATRIPLA as initial therapy. Of the 18 clinics where it was based on local decision, 18 stated feasibility as the reason for use (100 %), 1 (5.6 %) also stated it was a financial decision. Among the 15 clinics which stated they used ATRIPLA due to national guidelines, 14 clinics (93.3 %) indicated that this was due to feasibility, 4 clinics (26.7 %) also stated it was due to financial considerations (including 3 clinics who stated feasibility), and 1 clinic (6.7 %) stated it was due to efficacy. Of the 4 clinics which stated that ARTPLA was used as initial therapy for other reasons, these included that it was considered to be state-of-the-art therapy, financial reasons and in 2 clinics at the individuals request. A total of 30 clinics (85.7 %) stated ATRIPLA was used as a first-line regimen for feasibility either as part of local or national decision-making.

## Conclusion

Based on this survey of 96 clinics participating in EuroSIDA, it was apparent that a substantial proportion of clinics do not follow the recommended labelling for ATRIPLA, since 36.5 % of responding clinics use ATRIPLA as first-line therapy, primarily due to feasibility reasons. Six clinics, all in Eastern Europe, started the three components of ATRIPLA separately, and the observed regional differences are likely to some extent based on ATRIPLA availability and cost of the combination tablet in the Eastern European region.

One-third of participating clinics chose ATRIPLA as first-line antiretroviral therapy, despite the EMA licensing the regimen to HIV-positive persons with viral suppression for at least 3 months [3]. Of note, major international guidelines do not include a specific recommendation regarding this product information, but highlight the co-formulation of the agents and the availability of a single tablet regimen as an important advantage of this regimen [6–8]. In addition, results from 2012 to 2013 suggest that virologic response to ATRIPLA as first-line antiretroviral therapy, administered on an empty stomach, was similar to that of a first-line regimen of once-daily elvitegravir, cobicistat, emtricitabine and tenofovir, and was equally high in persons with baseline viral loads above 100,000 copies/ml [9, 10]. The difference between the cost of ATRIPLA and individual components also likely plays an important role in prescribing, but varies from country to country and even from clinic to clinic, likely depending on a number of factors including local contract negotiation between hospitals and pharmaceutical companies.

Limitations of this study include that not all clinics participated in the survey, although our response rate was >85 %. Centres participating in EuroSIDA tend to be centres of excellence and therefore may not be representative of all clinics in the European region. In addition, this survey was performed in 2012, and represents a cross-sectional survey of clinical practice at that time, which may since have changed. The major strength of this survey is the regional representation across Europe. We did not collect information on recommendations of the clinics as to when to take efavirenz and truvada when administered separately. Clinics may have advised persons to take efavirenz and truvada at night before bed to avoid twice-daily medication. Non-adherence to EMA guidelines may therefore be higher than shown in this report. Unfortunately, due to limited power, we were not able to compare the virological response following initiation of ATRIPLA as first-line antiretroviral therapy according to how it was initiated within the EuroSIDA study.

To conclude, over one-third of the participating clinics were using ATRIPLA as first-line antiretroviral therapy, despite recommendations that this regimen only be used in HIV-positive persons with >3 months virological suppression (<50 copies/ml) on their current regimen. Sites in many European countries report not adhering to the ATRIPLA summary of product characteristics in 2012, and the regulatory and legal implications to the individual sites or for individuals are unclear. Use of ATRIPLA was highest in Northern Europe, while Eastern and Southern Europe more commonly used truvada plus efavirenz. Main reasons cited include cost and availability.

## Appendix: The EuroSIDA Study Group

The multi-centre study group on EuroSIDA (national coordinators in parenthesis).

Argentina: (M. Losso), M. Kundro, Hospital JM Ramos Mejia, Buenos Aires.

Austria: (N. Vetter), Pulmologisches Zentrum der Stadt Wien, Vienna; R. Zangerle, Medical University Innsbruck, Innsbruck.

Belarus: (I. Karpov), A. Vassilenko, Belarus State Medical University, Minsk, V.M. Mitsura, Gomel State Medical University, Gomel; O. Suetnov, Regional AIDS Centre, Svetlogorsk.

Belgium: (N. Clumeck), S. De Wit, M. Delforge, Saint-Pierre Hospital, Brussels; R. Colebunders, Institute of Tropical Medicine, Antwerp; L Vandekerckhove, University Ziekenhuis Gent, Gent.

Bosnia-Herzegovina: (V Hadziosmanovic), Klinicki Centar Univerziteta Sarajevo, Sarajevo.

Bulgaria: (K Kostov), Infectious Diseases Hospital, Sofia.

Croatia: (J Begovac), University Hospital of Infectious Diseases, Zagreb.

Czech Republic: (L Machala), D Jilich, Faculty Hospital Bulovka, Prague; D Sedlacek, Charles University Hospital, Plzen.

Denmark: (J Nielsen), G Kronborg,T Benfield, M Larsen, Hvidovre Hospital, Copenhagen; J Gerstoft, T Katzenstein, A-B E Hansen, P Skinhøj, Rigshospitalet, Copenhagen; C Pedersen, Odense University Hospital, Odense; L Ostergaard, Skejby Hospital, Aarhus.

Estonia: (K Zilmer), West-Tallinn Central Hospital, Tallinn; Jelena Smidt, Nakkusosakond Siseklinik, Kohtla-Järve.

Finland: (M Ristola), Helsinki University Central Hospital, Helsinki.

France: (C Katlama), Hôpital de la Pitié-Salpétière, Paris; J-P Viard, Hôpital Necker-Enfants Malades, Paris; P-M Girard, Hospital Saint-Antoine, Paris; JM Livrozet, Hôpital Edouard Herriot, Lyon; P Vanhems, University Claude Bernard, Lyon; C Pradier, Hôpital de l’Archet, Nice; F Dabis, D Neau, Unité INSERM, Bordeaux.

Germany: (J Rockstroh), Universitäts Klinik Bonn; R Schmidt, Medizinische Hochschule Hannover; J van Lunzen, O Degen, University Medical Center Hamburg-Eppendorf, Infectious Diseases Unit, Hamburg; HJ Stellbrink, IPM Study Center, Hamburg; mM Bickel, JW Goethe University Hospital, Frankfurt; J Bogner, Medizinische Poliklinik, Munich; G. Fätkenheuer, Universität Köln, Cologne.

Greece: (J Kosmidis), P Gargalianos, G Xylomenos, J Perdios, Athens General Hospital; G Panos, A Filandras, E Karabatsaki, 1st IKA Hospital; H Sambatakou, Ippokration Genereal Hospital, Athens.

Hungary: (D Banhegyi), Szent Lásló Hospital, Budapest.

Ireland: (F Mulcahy), St. James’s Hospital, Dublin.

Israel: (I Yust), D Turner, M Burke, Ichilov Hospital, Tel Aviv; S Pollack, G Hassoun, Rambam Medical Center, Haifa; S Maayan, Hadassah University Hospital, Jerusalem.

Italy: (S Vella), Istituto Superiore di Sanità, Rome; R Esposito, I Mazeu, C Mussini, Università Modena, Modena; C Arici, Ospedale Riuniti, Bergamo; R Pristera, Ospedale Generale Regionale, Bolzano; F Mazzotta, A Gabbuti, Ospedale S Maria Annunziata, Firenze; V Vullo, M Lichtner, University di Roma la Sapienza, Rome; A Chirianni, E Montesarchio, M Gargiulo, Presidio Ospedaliero AD Cotugno, Monaldi Hospital, Napoli; G Antonucci, A Testa, G D`Offizi, C Vlassi, M Zaccarelli, A Antorini, Istituto Nazionale Malattie Infettive Lazzaro Spallanzani, Rome; A Lazzarin, A Castagna, N Gianotti, Ospedale San Raffaele, Milan; M Galli, A Ridolfo, Osp. L. Sacco, Milan; A d’Arminio Monforte, Istituto Di Clinica Malattie Infettive e Tropicale, Milan.

Latvia: (B Rozentale), I Zeltina, Infectology Centre of Latvia, Riga.

Lithuania: (S Chaplinskas), Lithuanian AIDS Centre, Vilnius.

Luxembourg: (T Staub), R Hemmer, Centre Hospitalier, Luxembourg.

Netherlands: (P Reiss), Academisch Medisch Centrum bij de Universiteit van Amsterdam, Amsterdam.

Norway: (V Ormaasen), A Maeland, J Bruun, Ullevål Hospital, Oslo.

Poland: (B Knysz) J Gasiorowski, Medical University, Wroclaw; A Horban, E Bakowska, Centrum Diagnostyki i Terapii AIDS, Warsaw; A Grzeszczuk, R Flisiak, Medical University, Bialystok; A Boron-Kaczmarska, M Pynka, M Parczewski, Medical Univesity, Szczecin; M Beniowski, E Mularska, Osrodek Diagnostyki i Terapii AIDS, Chorzow; H Trocha, Medical University, Gdansk; E Jablonowska, E Malolepsza, K Wojcik, Wojewodzki Szpital Specjalistyczny, Lodz.

Portugal: (F Antunes), M Doroana, L Caldeira, Hospital Santa Maria, Lisbon; K Mansinho, Hospital de Egas Moniz, Lisbon; F Maltez, Hospital Curry Cabral, Lisbon.

Romania: (D Duiculescu), Spitalul de Boli Infectioase si Tropicale: Dr. Victor Babes, Bucharest.

Russia: (A Rakhmanova), Medical Academy Botkin Hospital, St Petersburg; N Zakharova, St Petersburg AIDS Centre, St Peterburg; S Buzunova, Novgorod Centre for AIDS, Novgorod.

Serbia: (D Jevtovic), The Institute for Infectious and Tropical Diseases, Belgrade.

Slovakia: (M Mokráš), D Staneková, Dérer Hospital, Bratislava. Slovenia: (J Tomazic), University Clinical Centre Ljubljana, Ljubljana.

Spain: (J González-Lahoz), V Soriano, P Labarga, J Medrano, Hospital Carlos III, Madrid; S Moreno, J. M. Rodriguez, Hospital Ramon y Cajal, Madrid; B Clotet, A Jou, R Paredes, C Tural, J Puig, I Bravo, Hospital Germans Trias i Pujol, Badalona; JM Gatell, JM Miró, Hospital Clinic i Provincial, Barcelona; P Domingo, M Gutierrez, G Mateo, MA Sambeat, Hospital Sant Pau, Barcelona.

Sweden: (A Blaxhult), Venhaelsan-Sodersjukhuset, Stockholm; L Flamholc, Malmö University Hospital, Malmö; A Thalme, A Sonnerborg, Karolinska University Hospital, Stockholm.

Switzerland: (B Ledergerber), R Weber, University Hospital, Zürich; P Francioli, M Cavassini, Centre Hospitalier Universitaire Vaudois, Lausanne; B Hirschel, E Boffi, Hospital Cantonal Universitaire de Geneve, Geneve; H Furrer, Inselspital Bern, Bern; M Battegay, L Elzi, University Hospital Basel.

Ukraine: (E Kravchenko), N Chentsova, Kiev Centre for AIDS, Kiev; V Frolov, G Kutsyna, Luhansk State Medical University; Luhansk; S Servitskiy, Odessa Region AIDS Center, Odessa; M Krasnov, Kharkov State Medical University, Kharkov.

United Kingdom: (S Barton), St. Stephen’s Clinic, Chelsea and Westminster Hospital, London; AM Johnson, D Mercey, Royal Free and University College London Medical School, London (University College Campus); A Phillips, MA Johnson, A Mocroft, Royal Free and University College Medical School, London (Royal Free Campus); M Murphy, Medical College of Saint Bartholomew’s Hospital, London; J Weber, G Scullard, Imperial College School of Medicine at St. Mary’s, London; M Fisher, Royal Sussex County Hospital, Brighton; C Leen, Western General Hospital, Edinburgh.

Steering Committee: J Gatell, B Gazzard, A Horban, I Karpov, B Ledergerber, M Losso, A D’Arminio Monforte, C Pedersen, A Rakhmanova, M Ristola, J Rockstroh (Chair), S De Wit (Vice-Chair).

Additional voting members: J Lundgren, A Phillips, P Reiss.

Coordinating Centre Staff: O Kirk, A Mocroft, A Cozzi-Lepri, D Grint, A Schultze, L Shepherd, M Sabin, D Podlekareva, J Kjær, L Peters, J Nielsen, J Tverland, A H Fischer.

EuroSIDA representatives to EuroCoord: O Kirk, A Mocroft, J Grarup, P Reiss, A Cozzi-Lepri, R Thiebaut, J Rockstroh, D Burger, R Paredes, J Kjær. L Peters.
